# Immediate Dental Implants Enriched with L-PRF in the Esthetic Zone

**DOI:** 10.1155/2018/9867402

**Published:** 2018-12-03

**Authors:** Manoti Sehgal, Lovleen Puri, Sapna Yadav, Puja Malhotra, Sumit Singh Phukela, Bhupender Yadav, Bharti Raina

**Affiliations:** Department of Prosthodontics, Faculty of Dental Sciences, SGT University, Gurgaon, India

## Abstract

The aim of this article is to present the clinical application of immediate implant placement with L-PRF and immediate prosthetic loading in anterior esthetic region. A 24-year-old healthy female patient reported with a chief complaint of poor esthetics in the upper front tooth region with retained deciduous teeth. On oral examination, there were retained deciduous teeth (52, 53, and 63) with congenitally missing permanent successors. The retained deciduous teeth were extracted, and immediate implant placement was done in the extraction sockets along with L-PRF membranes in one surgical session under local anesthesia. Immediate temporization was performed with composite crowns on immediately placed dental implants. After 3 months of the healing period, the final implant-level impressions were made and the temporary composite crowns were replaced with the final zirconia porcelain crowns. A 12-month follow-up was made, and satisfactory esthetic and functional results were obtained.

## 1. Introduction

Retained primary teeth without permanent successors pose a great restorative challenge for the clinicians and cause functional and esthetic problems for patients. Compromised esthetics, shifting of adjacent teeth, altered occlusion, and supraeruptions of teeth are among the problems that can occur when a permanent tooth is congenitally missing. The mandibular second premolars are the most frequent congenitally missing permanent teeth followed in prevalence by the maxillary lateral incisors [1].

While various treatment approaches for congenitally missing teeth have been proposed, outcome data pertaining to these treatment options are lacking [2]. Replacement of a missing tooth with a dental implant offers specific advantages over other options for tooth replacement such as removable or fixed dentures [3]. These advantages include preservation of the alveolar crest, elimination of the need to restore adjacent teeth, and improved esthetics and function. By understanding the principles of treatment planning, implant surgery, and implant restoration, a clinician can successfully replace a retained primary tooth with a dental implant providing acceptable form, function, and esthetics to the patient.

Dental implantology has been extensively researched in basic and clinical grounds. In an effort to improve and accelerate healing of both hard and soft tissues in immediate implant placement, substitutes including growth factors and biomaterials have been traditionally employed. Membranes were also introduced to separate tissues. Recent research clearly indicates that L-PRF (leukocyte-platelet-rich fibrin, a second generation of platelet concentrates) significantly enhances wound healing in both soft and hard tissues [4–6].

To achieve better esthetic and functional results in the immediate implant placement technique, the use of L-PRF is beneficial at the osteotomy site. PRF consists of an autologous leukocyte-platelet-rich fibrin matrix composed of a tetra molecular structure, with cytokines, platelets, and stem cells within, which acts as a biodegradable scaffold, and favors the development of microvascularization and is able to guide epithelial cell migration to its surface [7, 8].

Some studies have demonstrated that PRF is a healing biomaterial with a great potential for bone and soft tissue regeneration, without inflammatory reactions, which may be used alone or in combination with bone grafts, promoting hemostasis, bone growth, and maturation [8, 9].

The aim of this study was to assess the clinical effectiveness of L-PRF in immediate implant placement in the anterior esthetic region along with immediate prosthetic loading.

## 2. Case Presentation

**Figure 1:**
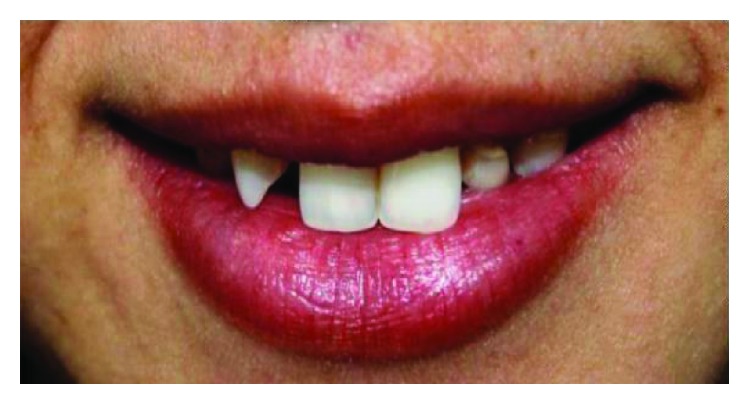
Preoperative smile view.

**Figure 2:**
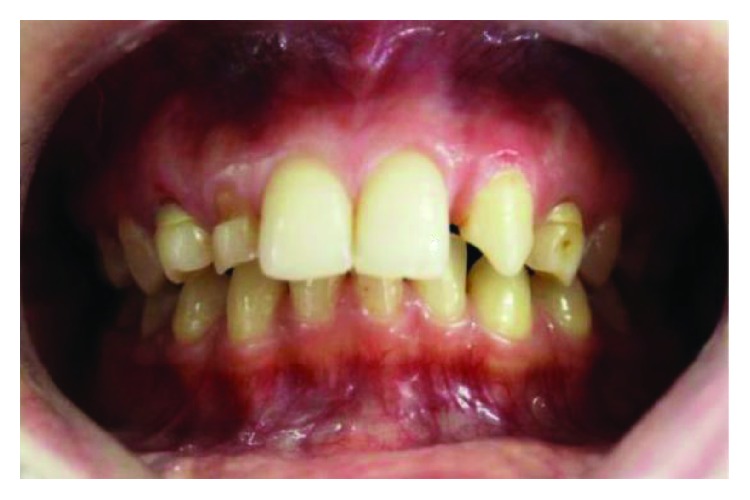
Preop intraoral frontal view.

A 24-year-old female patient in a good health condition without any chronic diseases reported to the Postgraduate Department of Prosthodontics, SGT University, Gurgaon, with a chief complaint of abnormal-looking upper front teeth since 10 years (Figure 1). On examination, the retained deciduous maxillary right lateral incisor and canine and deciduous left canine were observed along with the congenitally missing permanent maxillary left lateral incisor (Figure 2).

Since the teeth were present in the esthetic zone, the patient demanded a fixed prosthesis that would be both functionally and esthetically acceptable. After discussing all the possible treatment options with the patient, a treatment plan was formulated which included immediate implant placement following extraction wrt the deciduous teeth (52, 53, and 63) and fixed prosthesis following intentional endodontic therapy wrt 23.

**Figure 3:**
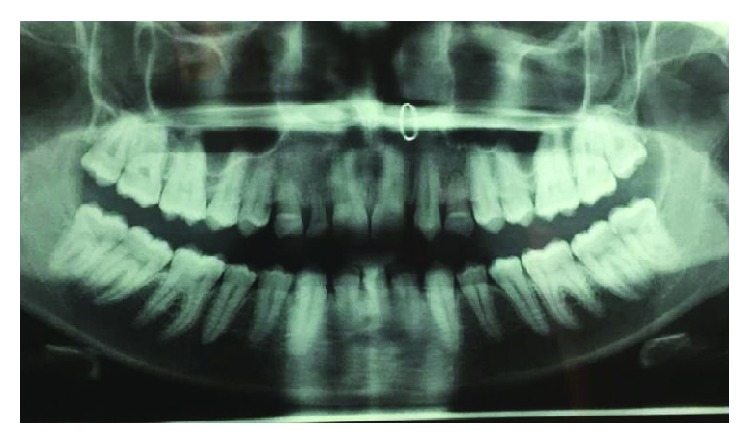
Preop OPG.

Diagnostic impressions were made, and the casts were poured. Preoperative orthopantomograph (Figure 3) and RVGs with respect to 52, 53, and 63 were taken. After completion of phase 1 therapy, a detailed case history was recorded, the proposed treatment plan was explained, and written informed consent was taken from the patient.

### 2.1. Surgical Procedure

**Figure 4:**
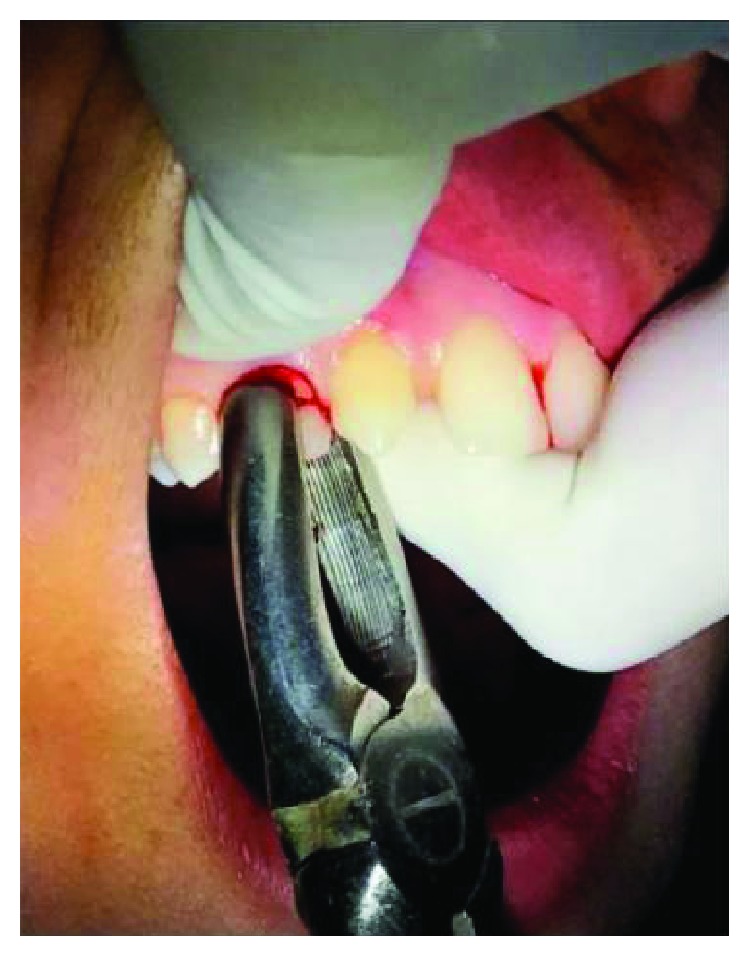
Atraumatic extraction.

**Figure 5:**
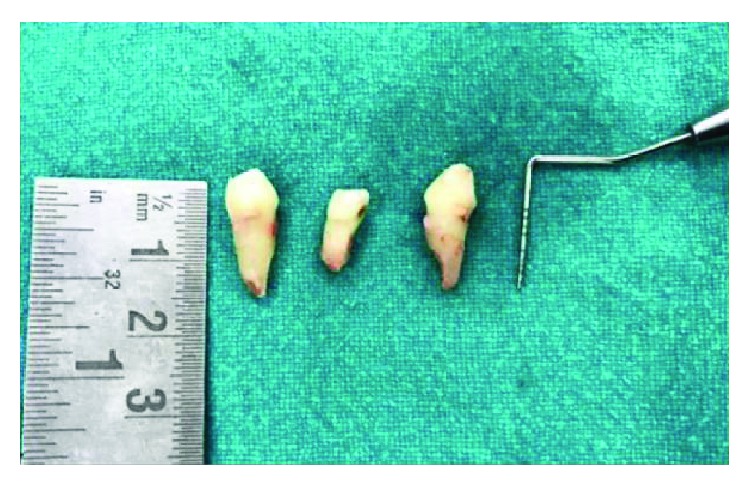
Root length measurements.

1 g Augmentin™, a combination of amoxicillin and clavulanate, was given to the patient one hour prior to the surgery as a part of prophylactic antibiotic therapy. Amoxicillin fights bacteria in the body. Clavulanate potassium is a beta-lactamase inhibitor that helps prevent certain bacteria from becoming resistant to amoxicillin. The patient was draped, and an extraoral scrub procedure with povidone-iodine solution was done as an asepsis protocol. After administration of local anesthesia, the procedure was performed atraumatically with the careful use of luxators (SDI®) and periotomes (Medessa®) to avoid damage of the continuity of the alveolar ridge and with anterior forceps (Figure 4) with minimal tissue damage to preserve the gingiva as well as the socket. The extraction sites were examined for the presence of any bony defect, and the root measurements of the extracted teeth were taken to decide the tentative implant sizes (Figure 5).

### 2.2. Preparation of the L-PRF Membrane

**Figure 6:**
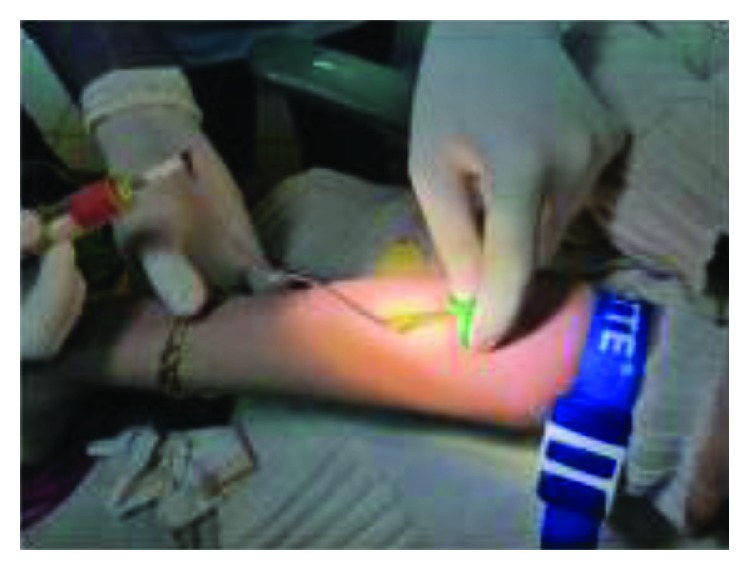
Withdrawing venous blood.

**Figure 7:**
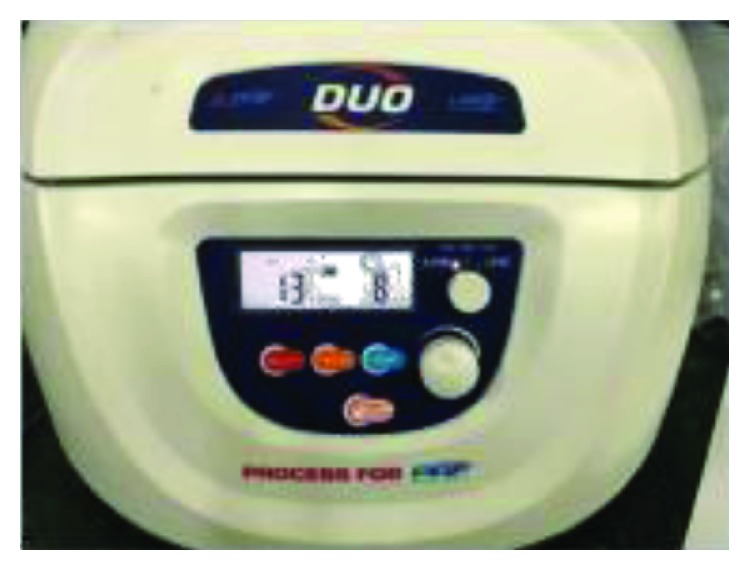
PRF machine.

**Figure 8:**
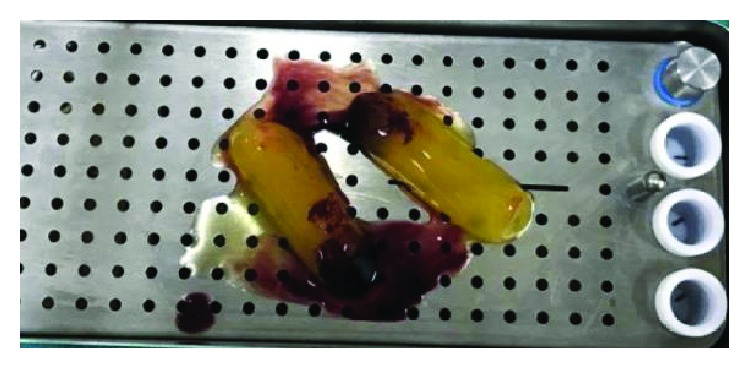
Preparation of the L-PRF membrane.

Ten milliliters of venous blood was withdrawn from the antecubital vein of the patient (Figure 6) and immediately centrifuged (PRF Duo™) at 3000 rpm for 13 min to obtain an L-PRF clot (Figure 7). The clot was condensed on a surgical plate and converted into a high-tensile-strength L-PRF membrane [10] (Figure 8). Leukocyte-platelet-rich fibrin (L-PRF) is a second generation of autologous platelet concentration and a fibrin mesh consisting of leukocytes, growth factors, proteins, and cytokines. L-PRF has advantages over PRP and PRGF by having a strong fibrin structure and not requiring any biochemical modification through bovine thrombin or anticoagulants. L-PRF has a very significant slow sustained release of key growth factors for at least 1 week and up to 28 days, which stimulates its environment for a significant time during early phases of wound healing. Because of its natural fibrin framework properties, growth factors can keep their activity for a relatively longer period and promote tissue regeneration faster.

### 2.3. Implant Placement

**Figure 9:**
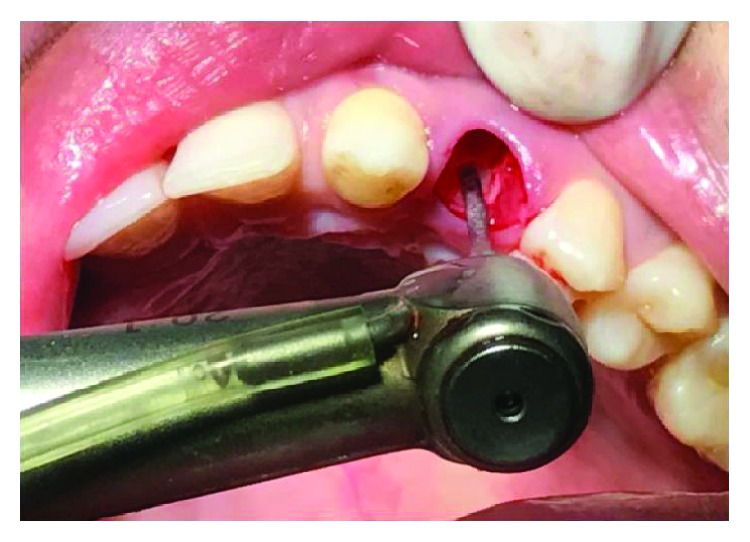
Apical and palatal preparation of the osteotomy site.

**Figure 10:**
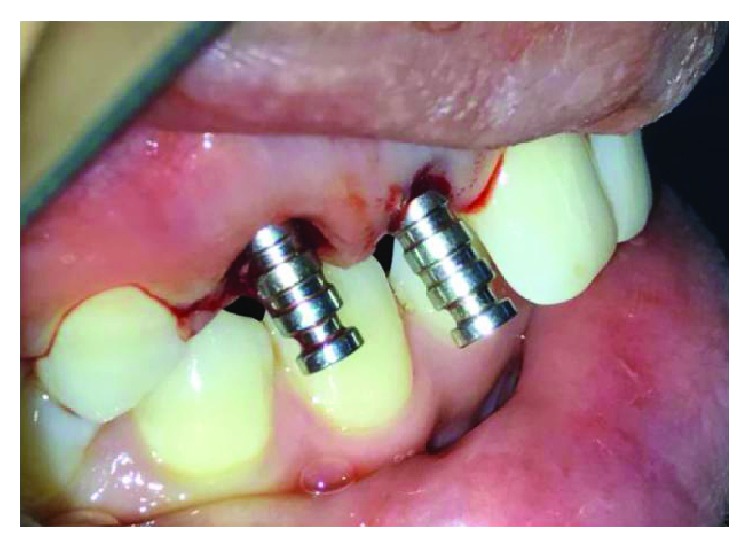
Palatal orientation of the osteotomy sites.

**Figure 11:**
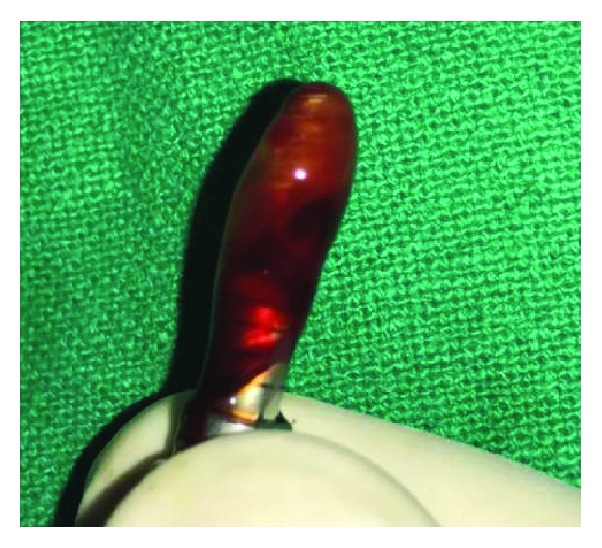
PRF gel-coated implant surface.

**Figure 12:**
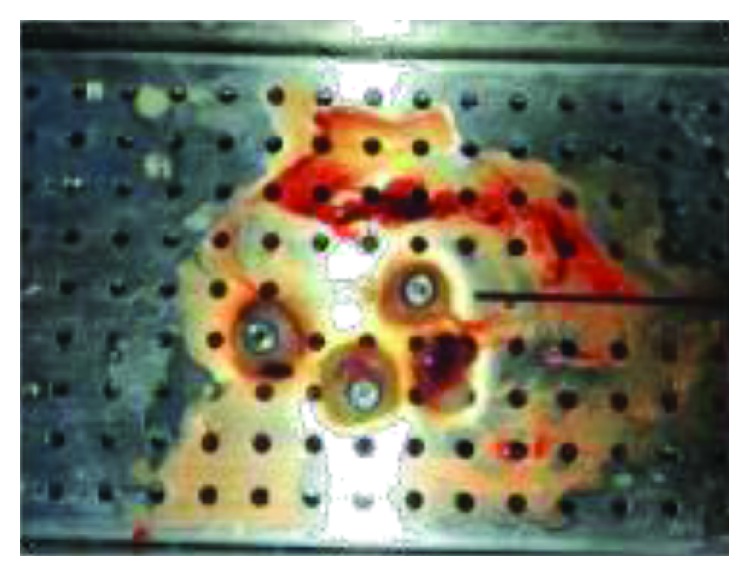
Poncho technique.

**Figure 13:**
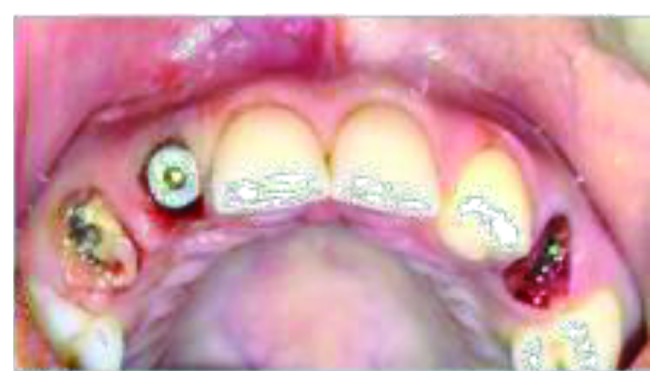
Final implant in placement.

**Figure 14:**
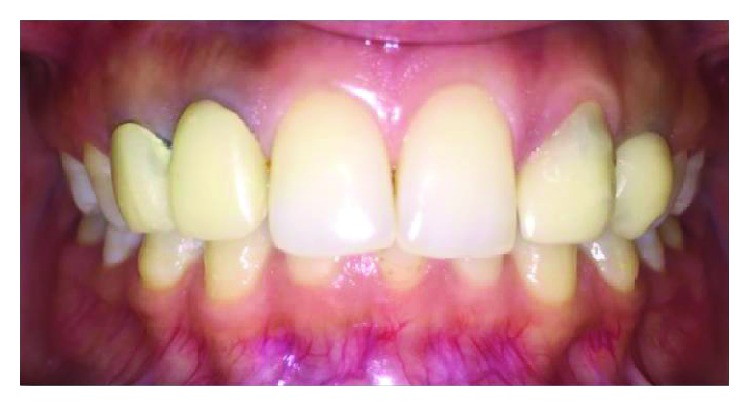
Temporization irt implants placed in positions of 53, 53, and 63.

The preparation of osteotomy sites was carried out using the sequential order of calibrated drills recommended by the manufacturer, cooled with saline solution in external mode at a speed of 800 rpm. The osteotomy sites were prepared on the palatal and apical aspects (Figure 9). The palatal orientation of the osteotomy sites was checked using paralleling pins (Figure 10). The implants used were screw-type, tapered-form, two-piece, and endosseous implants belonging to the Adin Touareg™ S implant system. The implants were inserted into the bone (with an insertion torque of 35–40 Ncm) using hand tools to achieve primary stabilization. The implant surfaces were coated with PRF gel (Figure 11). The implants were placed in the sockets, and cover screws were placed along with the L-PRF membrane using the poncho technique [11]. In this, PRF membranes were wrapped around the healing cap to favor soft tissue attachment and prevent infection and help to maintain the soft tissue profile (Figures 12 and 13). The poncho membranes can be placed around the implant collars to facilitate more rapid soft tissue healing without having to utilize a collagen barrier membrane. After ensuring the stabilization of the membrane, the final wash of the surgical site using povidone-iodine solution was done. Postoperative intraoral periapical radiograph was taken, confirming the accuracy of the placement of implants. Abutments were attached to the implant body and prepared for parallelism and adequate space. At the same day, provisional composite crowns were placed in the patient for immediate replacement of the missing front teeth due to functional and esthetic requirements. Meanwhile, intentional root canal therapy was done wrt 23. Following completion of the endodontic therapy, temporization was done wrt tooth no. 23 as well (Figure 14).

### 2.4. Postoperative Management

**Figure 15:**
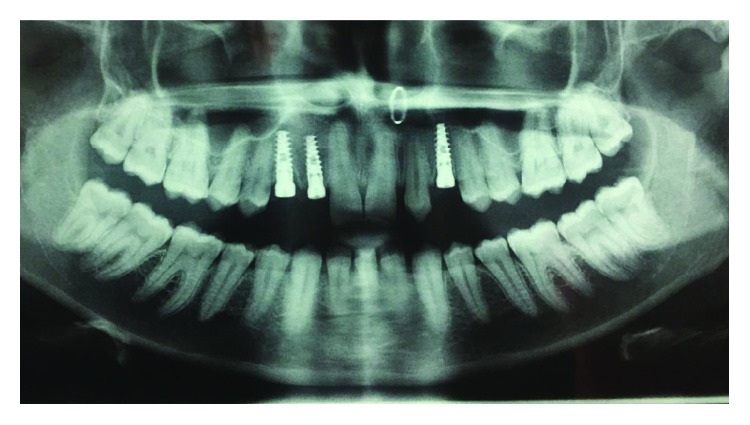
Postop OPG.

Postoperative care included soft diet and 0.12% chlorhexidine gluconate mouthwash twice daily starting from the next day. Systemic antibiotic and analgesic regimen including Augmentin (625 mg) TID for 5 days and the tablet Enzoflam D TID for 3 days were prescribed. A postoperative OPG was taken, and the patient was recalled after 1 week for follow-up (Figure 15).

### 2.5. Final Prosthesis and Follow-up

**Figure 16:**
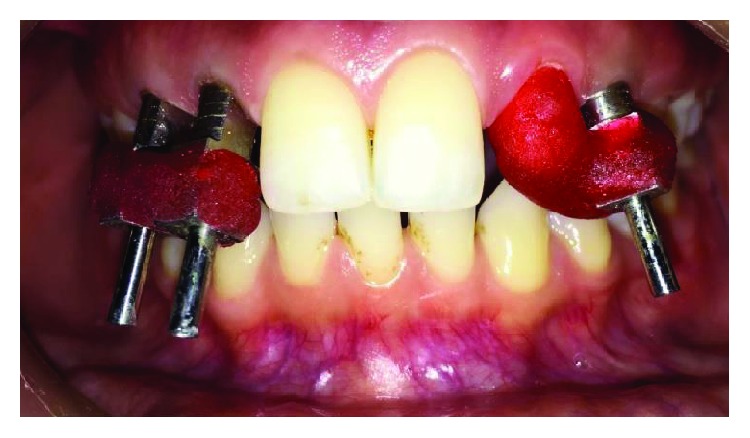
Jig trial to check the accuracy of impression.

**Figure 17:**
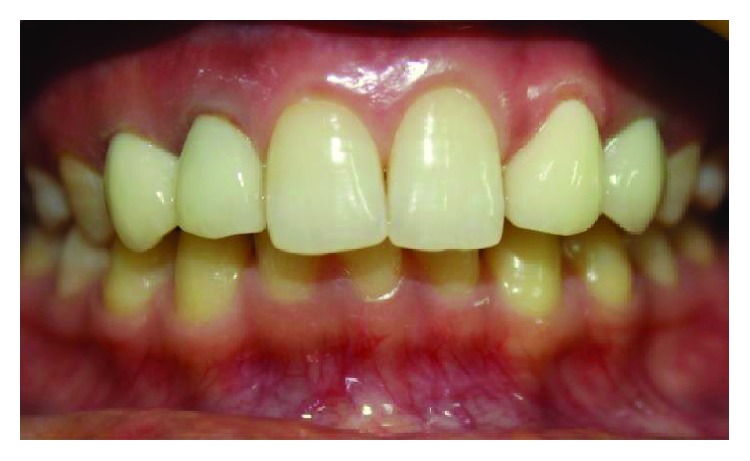
Final cementation of layered zirconia crowns.

**Figure 18:**
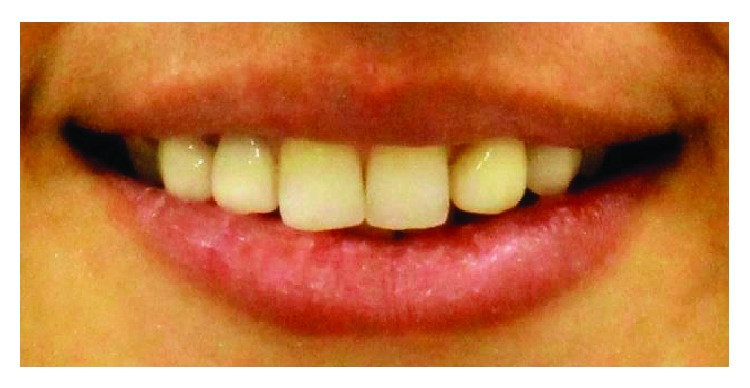
12 months of follow-up.

After a 3-month healing period, the patient was recalled for the definitive prosthesis. Radiographic evaluation revealed good osseointegration. The single-phase impression (3M™ ESPE™ (Soft) Monophase Polyether Impression Material) of implant transfers was made with an open tray technique, and a jig trial was done prior to prosthesis fabrication (Figure 16). The composite crowns were replaced with layered zirconia crowns and cemented with a resin cement (Calibra®, Dentsply Sirona) (Figure 17). Follow-up was done after 3-, 6-, and 12-month intervals (Figure 18). A comparison of pre- and postprocedure radiographs clearly revealed elevated peri-implant marginal bone in response to the action of loading forces. A very good esthetic result of this treatment was achieved by the preservation of gingival papillae.

## 3. Discussion

The patient with bilaterally retained maxillary deciduous canines and unilaterally retained deciduous lateral incisor was treated with an immediate implant with PRF associated with immediate nonfunctional loading. The healing period was uneventful, and no signs of implant mobility or peri-implant infection were reported during the evaluation period.

Traditionally, compromised teeth were removed and the resultant extraction sockets were left to heal for four to six months before dental implants were placed. However, marked alterations occur in the edentulous site following extraction, not only in the buccal-lingual/palatal dimension but also in the height of the buccal bone crest (decreased height). Improved implant hardware coupled with the patient's demand has shifted research focus towards shortened postextraction healing time or immediate implant placement following extraction. Reductions in the number of surgical interventions, a shorter treatment time, an ideal three-dimensional implant positioning, the presumptive preservation of alveolar bone at the side of the tooth extraction, and soft tissue esthetics have been claimed as the potential advantages of the immediate implant placement treatment approach [12].

Typically, implant stability and peri-implant tissue health are anticipated to decrease during the early weeks of healing; this is followed by an increase in implant stability. This is related to the biologic reactions of the bone and soft tissues to surgical trauma. Considering that hard and soft tissue modifications may cause esthetic and biological concerns, the planning of a protocol that improves the peri-implant tissue maintenance provides the best approach to an immediate postextractive implant placement procedure [13].

Growth factors represent a class of biologically active polypeptides that have a critical role in the healing process. Their use provides a new paradigm to understand the regenerative implantology [14]. Recently, platelet-based preparations from the patient's own blood have gained popularity in providing an inexpensive alternative to commercially available bioactive materials.

Leukocyte-platelet-rich fibrin (L-PRF) is a second generation of autologous platelet concentration and a fibrin mesh consisting of leukocytes, growth factors, proteins, and cytokines. L-PRF has advantages over PRP and PRGF by having a strong fibrin structure and not requiring any biochemical modification through bovine thrombin or anticoagulants. L-PRF has a very significant slow sustained release of key growth factors for at least 1 week and up to 28 days, which stimulates its environment for a significant time during early phases of wound healing. Because of its natural fibrin framework properties, growth factors can keep their activity for a relatively longer period and promote tissue regeneration faster [15].

Thus, modern clinical approaches that attempt to minimize tissue alteration after tooth extraction, while allowing for implant osseointegration in appropriate restorative positions for adequate function and esthetics, have been introduced and are currently under development.

## 4. Conclusion

In the present patient, immediate implants successfully replaced the unesthetic retained primary teeth. The use of PRF for the maintenance of crestal bone and soft tissue at the implant sites provided an adequate clinical condition for better esthetics associated with immediate implant placement. In certain situations, immediate implants may be considered a more appropriate alternative treatment of retained deciduous teeth than conventional implant therapy. Also, the use of platelet products like PRF and similar substrates can be employed in conjunction to immediate implants to further enhance the esthetics and postoperative healing. Clinical and radiographic evaluations after 12 months showed satisfactory preservation of marginal bone structure and peri-implant soft tissue condition as well as excellent esthetic rehabilitation which was highly accepted by the patient.
